# Anwendung fortgeschrittener Therapien bei chronisch-entzündlichen Darmerkrankungen

**DOI:** 10.1007/s00108-024-01833-w

**Published:** 2025-01-02

**Authors:** Benjamin Misselwitz, Sebastian Zeißig, Stefan Schreiber, Axel Dignass

**Affiliations:** 1 Medizinische Klinik und Poliklinik II, LMU München, München, Deutschland; 2 Klinik für Innere Medizin A, Universitätsmedizin Greifswald, Greifswald, Deutschland; 3 Klinik für Innere Medizin I, Universitätsklinikum Schleswig-Holstein, Kiel, Deutschland; 4 Medizinischen Klinik I, Agaplesion Markus Krankenhaus, Wilhelm-Epstein-Str. 4, 60431 Frankfurt/Main, Deutschland

**Keywords:** Januskinaseinhibitoren, Interleukininhibitoren, Sphingosin-1-Phosphat-Rezeptormodulatoren, Tumor-Nekrose-Faktor-Inhibitoren, Behandlungswirksamkeit, Janus kinase inhibitors, Interleukin inhibitors, Sphingosine-1-phosphate receptor modulators, Tumor necrosis factor inhibitors, Treatment efficacy

## Abstract

**Hintergrund:**

Die Therapieoptionen für chronisch-entzündliche Darmerkrankungen (CED) haben sich durch ein besseres Verständnis der zugrunde liegenden Pathogenese deutlich erweitert. Fünf Klassen fortgeschrittener Therapien stehen zur Verfügung.

**Ziel der Arbeit:**

Praxisnahe Übersicht über fortgeschrittene Therapien bei CED.

**Methoden:**

Narrativer Review.

**Ergebnisse und Diskussion:**

Fortgeschrittene Therapien sind bei mittel- bis schwerer CED indiziert. Der frühzeitige Einsatz wird empfohlen, um bessere Ansprechraten zu erreichen und eine irreversible, strukturelle Darmschädigung zu vermeiden. Tumor-Nekrose-Faktor(TNF)- und Januskinase(JAK)-Inhibitoren sind breit wirksam, auch bei extraintestinalen Krankheitsmanifestationen. Unter JAK-Inhibitoren ist das Risiko einer Varicella-zoster-Virus-Reaktivierung erhöht. Bei Hochrisikopatienten und im Alter > 65 Jahre besteht ein moderat erhöhtes Risiko kardiovaskulärer und neoplastischer Nebenwirkungen. Der Integrin-α4β7-Hemmer Vedolizumab und der Interleukin(IL)-12/-23-Inhibitor Ustekinumab haben ein sehr gutes Sicherheitsprofil. Selektive IL-23-Hemmer sind Ustekinumab bei vergleichbarem Sicherheitsprofil bezüglich der Wirksamkeit teilweise überlegen. Die Sphingosin-1-Phosphat-Rezeptormodulatoren Ozanimod und Etrasimod sind als orale Therapien bei Colitis ulcerosa zugelassen. Unverändert sind die Therapieerfolge der Medikamente begrenzt; auf jede individuelle Therapie wird eine Minderheit der Patienten nicht ansprechen. Dies erfordert oft die sequenzielle Gabe mehrerer Therapien. Aufgrund fehlender Vergleichsstudien erfolgen personalisierte Therapieauswahl, -sequenz und -entscheidung oft gemäß persönlicher Erfahrung; sie sollten u. a. Patientenpräferenz, Wirksamkeit, Sicherheit und individuelle Patientenprofile berücksichtigen.

## Abstract (en)

**Background:**

The treatment options for chronic inflammatory bowel diseases (IBD) have been greatly expanded due to a better understanding of the underlying pathogenesis. A total of five classes of advanced treatment are available.

**Objective:**

A practical overview of advanced treatment of IBD.

**Methods:**

Narrative review.

**Results and discussion:**

Advanced treatments are indicated for moderate to severe IBD. A timely use is recommended to achieve better response rates and to avoid irreversible bowel damage. Tumor necrosis factor (TNF) inhibitors and Janus kinase (JAK) inhibitors have a broad efficacy, also for extraintestinal disease manifestations. The risk of reactivation of varicella zoster virus is increased with JAK inhibitors. In high-risk patients and an age >65 years there is possibly a moderately elevated cardiovascular risk and neoplastic side effects. The integrin alpha4beta7 inhibitor vedolizumab and the interleukin (IL) 12 and 23 inhibitor ustekinumab have very good safety profiles. Selective IL-23 inhibitors are sometimes superior to ustekinumab with comparable safety profiles with respect to efficacy. The sphingosine-1-phosphate receptor modulators ozanimod and etrasimod are approved for oral treatment of ulcerative colitis. The treatment success of the medications remains still limited and a minority of patients will not respond to every individual treatment. Thus, sequential administration of several treatments is often needed. Due to the lack of comparative studies, the personalized choice, sequence and decision for treatments are usually based on personal experience and should take patient preferences, efficacy, safety and individual patient profiles into consideration.

Durch ein besseres Verständnis der Pathogenese chronisch-entzündlicher Darmerkrankungen (CED) konnten zielstrukturorientierte Behandlungsmöglichkeiten entwickelt werden, und eine Reihe dieser sog. fortgeschrittenen Therapien ist in den letzten Jahren zugelassen worden. Solche innovativen Therapien sind von konventionellen Therapien (5-Aminosalicylsäure, Steroide, Immunsuppressiva wie Azathioprin, 6‑Mercaptopurin, Methotrexat und Kalzineurininhibitoren) abzugrenzen. Aufgrund besserer Wirksamkeit und/oder erhöhter Sicherheit (für manche Klassen) haben sie einige konventionelle Therapien zurückgedrängt. Fortgeschrittene Therapien umfassen monoklonale Antikörper („Biologika“) und niedermolekulare Verbindungen („small molecules“). Die Vielfalt mit 5 unterschiedlichen zugelassenen Therapieklassen ist ein Segen für die Patienten, aber eine Herausforderung für die Behandler. Dieser Beitrag bietet einen Überblick über zielstrukturorientierte CED-Therapien und gibt Hinweise für ihre Anwendung [1–4].

## Grundlagen

### Therapeutische Schwelle

Alle CED-Medikamente teilen eine wichtige Limitation: Keine medikamentöse Strategie kann die große Mehrheit der Patienten (> 75 %) mit mittelschwerer bis schwerer Erkrankung nachhaltig in eine klinische Remission bringen und in ihr halten. In Zulassungsstudien (Tab. 1) betragen die klinischen Remissionsraten 20–50 % gegenüber 5–30 % bei Placebo, und bei etwa 30 % der Patienten kann eine endoskopische Remission erreicht werden. In der klinischen Praxis und in „Real-world“-Studien sind die Ansprechraten höher als in einem stringenten Studiendesign. Dennoch ist bei vielen Patienten die sequenzielle empirische Anwendung unterschiedlicher Wirkstoffklassen notwendig. Um diese therapeutische Schwelle („therapeutic ceiling“) zu durchbrechen, werden wirksamere Medikamente bzw. klare klinische Prädiktoren oder Biomarker für eine Vorhersage des individuellen Therapieansprechens benötigt.

**Tab. 1:** Fortgeschrittene Therapien bei chronisch-entzündlichen Darmerkrankungen (*CED*)

Wirkstoff	Indikation (EMA)	Zulassungsjahr (EMA)	Dosierung	Intensivierte Dosis	Biosimilar, Generika	Zulassungsstudien
**TNF-Inhibitoren**						
Infliximab	CU, MC PS, PA, RA, AS	1998 für MC 2005 für CU	5 mg/kgKG i.v. Wo 0, 2, 6, dann alle 8 Wo *Oder*: ab Wo 6 120 mg s.c. alle 2 Wochen	10 mg/kgKG .i.v. alle 4 Wo, 120 mg s.c./Wo	Ja	[36–38]
Adalimumab	CU, MC PS, PA, RA, AS, PJIA, AERA, UV	2007 für MC 2011 für CU	*MC*: 80 mg s.c. Wo 0, dann 40 mg alle 2 Wo *CU*: 160 mg s.c. Wo 0, 80 mg Wo 2, dann 40 mg alle 2 Wo	40 mg s.c./Wo	Ja	[39]
Golimumab	CU PA, RA, AS, PJIA	2013 für CU	200 mg Wo 0, 100 mg Wo 2, 50–100 mg alle 4 Wo	100 mg s.c. alle 4 Wo	Nein	[40]
**Integrininhibitoren**						
Vedolizumab	CU, MC	2014 für CU 2014 für MC	300 mg i.v. Wo 0, 2, 6, dann alle 8 Wo 300 mg i.v. *Oder*: ab Wo 6 108 mg alle 2 Wo s.c.	Ab Wo 6 300 mg alle 4 Wo i.v. oder	Nein	[41, 42]
**Interleukin-12/-23-Hemmer**						
Ustekinumab	CU, MC PS, PA	2016 für MC 2019 für CU	Gewichtsadaptiert 260 mg/390/520 mg, i.v., dann 90 mg alle 8–12 Wo s.c.	Off-label Anwendung 90 mg alle 4–6 Wo s.c.	Ja (MC) Nein (CU)	[43, 44]
**Selektive Interleukin-23-Hemmer**						
Risankizumab	MC, CU PS, PA	2022 für MC 2024 für CU	*MC*: 600 mg i.v. Wo 0, 4, 8, dann 360 mg s.c. Wo 12 und alle 8 Wo danach *CU*: 1200 mg i.v. Wo 0, 4, 8, dann 180/360 mg s.c Wo 12 und alle 8 Wo danach	–	Nein	[45, 46]
Mirikizumab	CU	2023 für CU	300 mg i.v., Wo 0, 4, 8, (ggf. verlängert zusätzlich Wo 12, 16, 20), dann 200 mg alle 4 Wo s.c.	–	Nein	[47]
Guselkumab	PS, PA	Ausstehend	Noch offen	–	Nein	–
**JAK-Inhibitoren**						
Tofacitinib	CU RA, PA	2018 für CU	2‑mal 10 mg p.o. für 8–16 Wochen, dann 2‑mal 5 mg p.o.	2‑mal 10 mg p.o.	Nein	[27]
Upadacitinib	CU, MC RA, PA, AS, AD	2022 für CU 2023 für MC	CU: 45 mg p.o. für 8–16 Wo, dann 15 mg p.o. MC: 45 mg p.o. für 12 Wo, dann 15 mg p.o.	30 mg p.o.^a^	Nein	[26, 28]
Filgotinib	CU, RA	2021 für CU	200 mg einmal tgl.^b^	–	Nein	[29]
**Sphingosin-1-Phosphat-Rezeptormodulatoren**						
Ozanimod	CU RRMS	2021 für CU	0,92 mg einmal tgl. p.o. Einschleichende Dosierung Tage 1–7	–	Nein	[48]
Etrasimod	CU	2023 für CU	2 mg einmal tgl. p.o.	–	Nein	[49]

*AD* atopische Dermatitis, *AERA* Active Enthesitis-Related Arthritis, *AS* ankylosierende Spondylarthritis, *CU* Colitis ulcerosa, *JAK* Januskinase, *MC* Morbus Crohn, *PA* Psoriasis-Arthritis, *PJIA* polyartikuläre juvenile idiopathische Arthritis, *PS* Plaque-Psoriasis, *RA* rheumatoide Arthritis, *RRMS* Relapsing-Remitting Multiple Sclerosis, *TNF* Tumor-Nekrose-Faktor, *UV* nichtinfektiöse Uveitis, *Wo* Woche/Wochen

^a^Bei Patienten > 65 Jahren nicht empfohlen

^b^Bei Risikofaktoren 100 mg einmal tgl.

### (Fehlende) Unterschiede zwischen M. Crohn und Colitis ulcerosa

Morbus Crohn (MC) und Colitis ulcerosa (CU) besitzen eine Reihe von klinischen und therapeutischen Gemeinsamkeiten, sie sind aber oft klinisch unterscheidbare Krankheiten, obwohl die moderne Genetik eher auf ein Kontinuum hinweist, an deren Extremen der reine MC des Ileums bzw. die klassische CU stehen [5]. Daher ist für jedes neue CED-Medikament eine separate Wirksamkeitsprüfung und Zulassung für MC und CU erforderlich (Tab. 1). Dennoch scheint die Mehrheit der zugelassenen Substanzklassen bei beiden Krankheiten wirksam zu sein, obwohl sich die Dosierungen und die relative Wirksamkeit unterscheiden können. Eine mögliche Ausnahme stellen Mesalazine (5-ASA-Präparat) dar; diese erreichen das Gewebe nach oraler oder rektaler Gabe vom Lumen her und wirken möglicherweise bei CU mit oberflächlicher Entzündung besser als bei MC mit einer transmuralen Entzündung.

#### Merke

Die Mehrheit der zugelassenen Substanzklassen scheint sowohl bei MC als auch bei CU wirksam zu sein, obwohl sich die Dosierungen und die relative Wirksamkeit unterscheiden können.

### Therapiebeginn

Eine fortgeschrittene Therapie ist bei CED mit mittelschwerer oder schwerer Erkrankung nach Versagen oder Unverträglichkeit der konventionellen Therapie indiziert. Eine mittlere Schwere der Erkrankung ist nicht einheitlich und teilweise unscharf definiert, und es müssen Beschwerden, endoskopischer Befund, klinische Präsentation (Stenosen, Fisteln, Befall des oberen Gastrointestinaltrakts), klinischer Verlauf (z. B. Steroidabhängigkeit, Steroidrefraktärität, Hospitalisationsbedürftigkeit) und extraintestinale Manifestationen (EIM) berücksichtigt werden [1–3, 6].

#### Merke

Eine fortgeschrittene Therapie ist bei CED mit mittelschwerer oder schwerer Erkrankung nach Versagen oder Unverträglichkeit der konventionellen Therapie indiziert.

Das zweite Kriterium, Versagen oder Unverträglichkeit der konventionellen Therapie, ist unter Diskussion: Im Allgemeinen und im Verständnis der Zulassungsbehörden wird hier ein Versagen auf eine Therapie von entweder Steroiden, Immunmodulatoren oder 5‑ASA-Präparaten verstanden; ein Therapieversuch mit Azathioprin ist nicht obligat.

Eine aggressive Therapiestrategie („hit early, hit hard“) wird zunehmend durch klinische Evidenz untermauert. In der aktuellen PROFILE-Studie war bei neu diagnostiziertem MC eine „Top-down-Strategie“ (sofortiger Start der Gaben von Infliximab + Azathioprin) einer „Accelerated-step-up-Strategie“ mit initialer klassischer Steroidtherapie, der Verabreichung von Azathioprin beim 1. Rezidiv und Azathioprin + Infliximab beim 2. Rezidiv klar überlegen [7]. Eine frühe aggressive Strategie soll einer Beschädigung des Gastrointestinaltrakts durch lange Entzündung mit konsekutiver irreversibler Stenosierung, Fistulierung und Operationen vorbeugen. Eine lange unbehandelte Entzündung ist ebenfalls mit dem Risiko von Komplikationen (z. B. Malignität, Thrombosen, Herz-Kreislauf-Erkrankungen) assoziiert und spricht im späteren Krankheitsverlauf nur seltener und unvollständiger auf medikamentöse Behandlungen an.

Auf der anderen Seite kann die Erstpräsentation einer CED mit einer passageren (z. B. infektiösen) Enteritis verwechselt werden, und eine sehr frühe „Top-down-Strategie“ resultiert in einer „erfolgreichen“, aber unnötigen langjährigen Behandlung bei einem Teil der Patienten. In Abwesenheit klarer Kriterien für einen schweren Verlauf kann mit dem Beginn einer fortgeschrittenen Therapie für kurze Zeit zugewartet werden (Steroid-Tapering, Therapieversuch mit 5‑ASA bei CU). Bei persistierender oder erneuter Krankheitsaktivität sollte die fortgeschrittene Therapie jedoch nicht hinausgezögert werden.

### Wahl der antientzündlichen Therapie

**Abb. 1:**
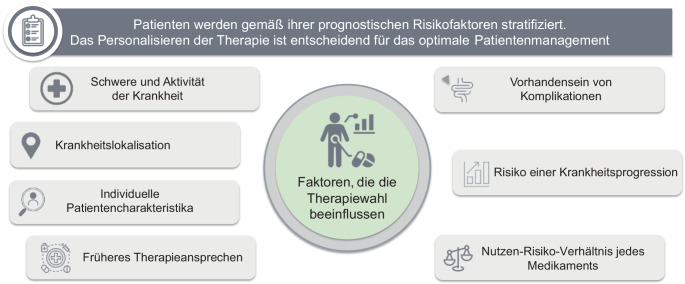
Faktoren, die die Wahl einer fortgeschrittenen Therapie beeinflussen. (Mod. nach [1–3])

**Abb. 2:**
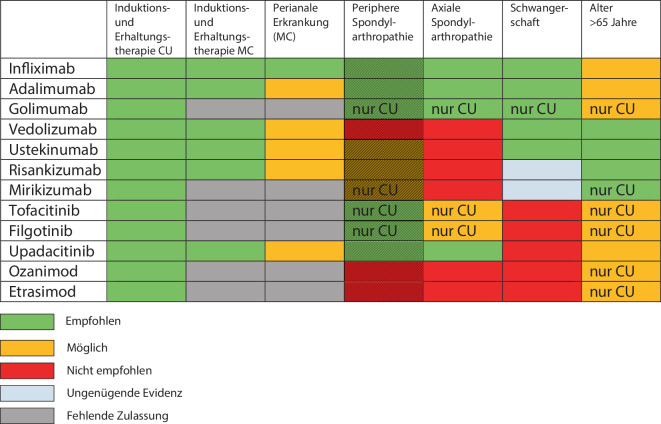
Einsatzmöglichkeiten fortgeschrittener Therapien bei chronisch-entzündlichen Darmerkrankungen, teilweise übernommen aus Raine et al. [2]. Die Abbildung gibt die Meinung der Autoren grob vereinfacht wieder, und die Indikation sollte individuell sorgfältig geprüft werden. Bei peripherer Spondylarthropathie ist die Evidenz insgesamt ungenügend (durch die *Schattierung* angedeutet). *CU* Colitis ulcerosa, *MC* Morbus Crohn

Grundsätzlich sind alle fortgeschrittenen medikamentösen Therapien nach Versagen oder Unverträglichkeit der konventionellen Therapie bei therapienaiven (ohne vorherige fortgeschrittene Therapie) und mit fortgeschrittener Therapie erfahrenen Patienten zugelassen. Jedes der in Tab. 1 aufgeführten Medikamente kann in erster und jeder weiteren Therapielinie gegeben werden. Kriterien für die Therapiewahl sind u. a.: Wirksamkeit, Sicherheit, Vortherapien, Schwangerschaftswunsch, Stillzeit, Komorbiditäten und individuelle Risiken (Tab. 2; Abb. 1 und 2), Applikationsform (p.o., s.c., i.v.), Erfahrung (für den jeweiligen Behandler bzw. global Patientenjahre), Schnelligkeit des Ansprechens, Wirksamkeit bei EIM und Fisteln und Medikamentenpreis. Nicht selten stehen mehrere geeignete Medikamente zur Verfügung (Abb. 2).

**Tab. 2:** Vorabklärungen, Kontraindikationen, Vorbereitung und unerwünschte Arzneimittelwirkungen (*UAW*) einer fortgeschrittenen Therapie

	Vorabklärungen, Vorbereitung	Wichtige Kontraindikationen	Wichtige UAW
Alle fortgeschrittenen Therapien	Hepatitis-B- und Hepatitis-C-Status (Hbs, Anti-Hbs‑, Anti-HBc‑, Anti-HCV-Antikörper), HIV-Status, Interferon-Gamma-Release Assay (Tuberkulose) Vervollständigen des Impfstatus^a,b^, Pneumokokken-, jährlich Corona‑, Grippeimpfung, jährliches Hautkrebsscreening Patientenaufklärung	Aktive Infektionen (z. B. aktive Hepatitis B, latente Tuberkulose), geplante Lebendimpfungen (z. B. Masern, Mumps Röteln) Vorsicht bei bereits bestehender Immunsuppression	Allergische Reaktionen, Infusions‑/Injektionsreaktionen
TNF-Hemmer	–	Herzinsuffizienz der NYHA-Stadien III und IV, Leberzirrhose des Child-Pugh-Score B/C, demyelinisierende ZNS-Erkrankungen	Infektiologische Komplikationen wie Reaktivierung von Tuberkulose, Hepatitis B, paradoxe Psoriasis, Immunphänomena (medikamenteninduzierter Lupus erythematodes), Verschlechterung einer Herzinsuffizienz, nichtinfektiöse Hepatitis
Integrinhemmer	–	–	Selten: Atemwegsinfektionen, Husten, gastrointestinale Infektionen, Arthralgien, Müdigkeit
IL-12/-23-, selektive IL-23-Hemmer	–	–	Auf Placeboniveau
JAK-Inhibitoren	Erfassen kardiovaskulärer Risikofaktoren, Malignomanamnese, regelmäßig Differenzialblutbild, Lipid‑, Leberwerte	Schwangerschaft, Stillen	Infektiologische Komplikationen (signifikant Varicella-zoster-Virus), Non-Melanoma Skin Cancer, Leberwerterhöhung, Malignome, kardiovaskuläre und thrombembolische Komplikationen
Sphingosin-1-Phosphat-Rezeptormodulatoren	EKG, kardiale Anamnese (ggf. weitere Abklärungen), Augenarztbesuch, regelmäßig Differenzialblutbild	Schwangerschaft, Stillen, kardiale Vorerkrankungen, zerebrovaskuläre Erkrankungen, Makulaödem	Lymphopenie, leichte infektiologische Komplikationen, AV-Block-Bilder (selten)

*AV* atrioventrikulär, *HBc* „hepatitis B core-antigen“. *HBs* „hepatitis B surface antigen“, *HCV* Hepatitis-C-Virus, *HIV* „human immunodeficiency virus“, *IL* Interleukin, *JAK* Januskinase, *NYHA* Klassifikation der New York Heart Association, *TNF* Tumor-Nekrose-Faktor, *ZNS* Zentralnervensystem

^a^Varicella-zoster-Impfung (Totimpfstoff) altersunabhängig vor JAK-Inhibitor-Therapie (starke Empfehlung der Autoren) sowie vor TNF-Inhibitor-Therapie (schwache Empfehlung). Das Robert Koch-Institut empfiehlt eine Varicella-zoster-Impfung bei gesunden Personen > 60 Jahren und bei chronisch kranken Patienten mit erhöhtem Zoster-Risiko ab 50 Jahren (https://www.rki.de/DE/Content/Kommissionen/STIKO/Empfehlungen/Impfempfehlungen_node.html)

^b^Eine Impfung sollte den Beginn einer fortgeschrittenen Therapie nicht verzögern

Um eine Patientenakzeptanz für jahrelange Behandlungen zu erreichen, müssen Entscheidungen von Arzt und Patient gemeinsam getroffen werden. Es ist unrealistisch, alle Einzelpräparate zu diskutieren, sondern generelle Patientenpräferenzen bezüglich Effektivität, Sicherheit und Applikationsformen sollten besprochen werden [8].

### Biologika und Biosimilars

Biologika sind biotechnologisch hergestellte monoklonale Antikörper oder Fusionsproteine. Nach Ablauf einiger Patentrechte sind jetzt generische Produkte, „Biosimilars“, verfügbar (Tab. 1). Die Markteinführung von Generika geht mit Preissenkungen (auch bei Originalpräparaten) und Kostenersparnissen einher, und es ist anzunehmen, dass so Akzeptanz und leitliniengerechter Einsatz der fortgeschrittenen Therapien verbessert werden.

Angesichts der komplexen Struktur von Antikörpern (Glykosylierungen, Proteinfaltung) und aufwendigen Reinigungsverfahren ist die Bioäquivalenz nicht notwendigerweise gegeben. Daher muss vor der Zulassung jedes Biosimilars die Gleichwertigkeit gegenüber dem Originalpräparat für eine Indikation (jedoch nicht zwangsläufig für CED) nachgewiesen werden. Für Infliximab konnte in einer großen randomisierten verblindeten Studie in einem gemischten Patientenkollektiv (Nor-Switch-Studie, 51 % CED-Patienten) gezeigt werden, dass ein Wechsel vom Originalpräparat auf ein Biosimilar die Krankheit nicht verschlechtert [9]. Gemäß unserem Wissenstand gibt es bisher keine Berichte über systematisch schlechtere Therapieergebnisse unter der Anwendung von Biosimilars [1]. Bei Patienten, die lange auf ein Originalpräparat eingestellt gewesen sind, kann die Weitergabe im Einzelfall aber sinnvoll sein, um Nocebo-Effekte zu vermeiden.

Innovative Biosimilars werden gelegentlich auch als Biobetter bezeichnet. Für Infliximab wurde eine s.c.- Applikationsform nur für ein Generikum entwickelt. Im Vergleich mit der i.v.-Gabe hatte das s.c.-Biosimilar deutlich höhere Medikamentenspiegel als das Originalpräparat gezeigt, bei einem nichtsignifikanten Trend zu besserer Wirksamkeit [10], aber möglicherweise niedrigerer Immunogenität und geringem Wirkverlust, sodass auch eine Monotherapie ohne Azathioprin mit hoher Effektivität und weniger Nebenwirkungen möglich ist. Manche Biosimilars weisen auch eine längere Haltbarkeit bei Raumtemperatur auf, was die Handhabung für Patienten vereinfacht.

### Biologikaspiegel und Anti-Drug-Antikörper

Bei CED-Patienten mit aktiver Entzündung besteht eine gestörte Darmbarriere mit Verlust der Biologika und anderer Proteine in das Darmlumen. Somit ist das Erreichen eines adäquaten Plasmaspiegels relevant, um den Circulus vitiosus von Entzündung, gestörter Darmbarriere, niedrigen Medikamentenspiegeln und schlechter Medikamentenwirkung zu durchbrechen. Ein Messen der Antikörperspiegel ist jedoch nur bei fehlender Medikamentenwirkung bzw. Wirkverlust sinnvoll, und valide minimale Talspiegel sind nur bei Tumor-Nekrose-Faktor(TNF)-Inhibitoren etabliert. In diesem Fall kann die Dosis erhöht oder das Intervall verkürzt (Tab. 1) bzw. eine Reinduktion durchgeführt werden.

Bei Vedolizumab könnten sehr hohe Talspiegel weniger effizient sein, denn in Fällen hoher Spiegel wurde neben der erwünschten Blockade der Einwanderung proinflammatorischer T‑Zellen auch eine Blockade der Migration antiinflammatorischer regulatorischer T‑Zellen (T_reg_) in den Darm beobachtet [11]. Für Mirikizumab und Risankizumab machen die verwendeten, sehr hohen Medikamentendosierungen Spiegelbestimmungen und Dosisintensivierungen wahrscheinlich obsolet.

Antikörper gegen Medikamente (Anti-Drug-Antikörper, ADA) sind aufgrund der unvollständigen Humanisierung (25 % Maussequenzen) besonders bei Infliximab in höherem Ausmaß zu erwarten (ca. 28 %), bei anderen Biologika seltener (ca. 4–8 %; [12]). Das Risiko einer ADA-Bildung kann bei Infliximab durch die Gabe von Azathioprin reduziert werden (ca. −50 % für Infliximab, > 50 % für andere Biologika; [12]). Die Kombination von Infliximab und Azathioprin für 6 bis 12 Monate ist eines der wenigen Beispiele einer nachgewiesenen synergistischen Wirkung von 2 CED-Medikamenten [13].

## Therapieklassen

### Tumor-Nekrose-Faktor-Inhibitoren (Infliximab, Adalimumab, Golimumab)

Infliximab war das erste von der European Medical Agency (EMA) bei CED zugelassene Biologikum (1998 für MC, 2005 für CU). Adalimumab ist sehr breit eingesetzt worden und für lange Zeit weltweit das umsatzstärkste immunologische Therapeutikum gewesen. Damit kann auf einen breiten Erfahrungsschatz zurückgegriffen werden.

Die TNF-Inhibitoren sind Biologika der Wahl zur Behandlung von einigen EIM und Fisteln. Sie sind ebenfalls für rheumatologische Indikationen und Uveitis zugelassen (Tab. 1). Für die Wirksamkeit bei kutanen EIM (Pyoderma gangraenosum) gibt es ebenfalls Fallserien [14], und die Wirksamkeit von Infliximab bei perianalen Fisteln wurde in randomisierten Studien gezeigt [15, 16].

Unerwünschte Arzneimittelwirkungen (UAW) der TNF-Inhibitoren sind infektiologische Komplikationen, wie eine Reaktivierung einer latenten Tuberkulose, die fulminant sein kann, oder eine Hepatitis-B-Reaktivierung. Opportunistische Infektionen sind in ihrer Häufigkeit knapp 2fach erhöht [17]. Weitere UAW sind demyelinisierende Erkrankungen (multiple Sklerose, Optikusneuritis), eine Dekompensation einer Herzinsuffizienz und immunologische Komplikationen (z. B. Drug-induced Lupus erythematodes, Risiko 0,2 %). Die TNF-Inhibitoren werden als Medikamente gegen Psoriasis eingesetzt, dennoch kommt es in etwa 5 % der Fälle zum Neuauftreten einer paradoxen Psoriasis unter Anti-TNF-Therapie.

Entgegen initialen Beobachtungen scheint das Risiko solider Tumoren unter Anti-TNF-Therapie nicht erhöht zu sein. Wegen der inkonsistenten Datenlage bezüglich Lymphomen und Melanomen bleibt ein gering erhöhtes Risiko für diese Erkrankungen möglich [18]. Bei einer kombinierten Therapie (z. B. Azathioprin + TNF-Blocker) ist das Risiko hämatologischer Malignome jedoch deutlich erhöht, v. a. bei einer Therapiedauer länger als ein  Jahr [18]. Gefürchtet werden schwer therapierbare hepatosplenische T‑Zell-Lymphome bei Männern < 30 Jahre. Insgesamt sollten die UAW in Relation zum langen und zum breiten Einsatz der TNF-Inhibitoren, der neue UAW unwahrscheinlich macht, und der breiten Wirksamkeit gewertet werden.

#### Merke

Die TNF-Inhibitoren stellen einen Goldstandard in der Therapie des fistulierenden Morbus Crohn und bei extraintestinalen Manifestationen von CED sowie bei fulminanten Erkrankungsverläufen dar. Die UAW der TNF-Inhibitoren sollten in Relation zu ihrem durch guten Kenntnisstand langen und breiten Einsatz sowie ihrer breiten Wirksamkeit gewertet werden.

### Anti-Integrine (Vedolizumab)

**Abb. 3:**
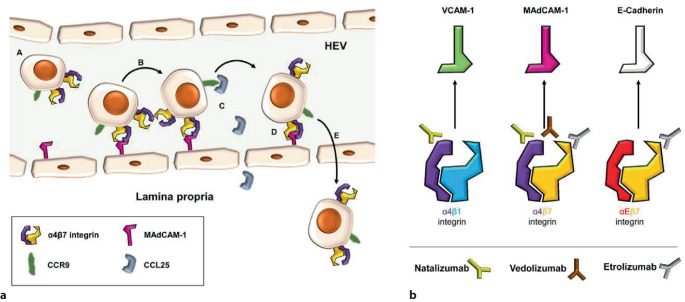
Wirkungsmechanismus der Integrininhibitoren. **a** Immunzellen binden über MadCAM-1 an Integrin-α4β7 und durchqueren das Endothel der Venolen im Magen-Darm-Trakt („Homing“). **b** Vedolizumab blockiert Integrin-α4β7 und hemmt das darmspezifische Homing von Immunzellen. Natalizumab bewirkt eine Integrin-α4-Blockade mit zusätzlichen Effekten auf das Homing in das Zentralnervensystem (über Integrin-α4β1) und dem Risiko zerebraler Infektionen. Etrolizumab bindet an Integrin-β7 mit zusätzlicher E‑Cadherin-Blockade, ist in klinischen Studien bei CED jedoch wirkungslos geblieben. *CCL25* Chemokine (C-C Motif) Ligando 25, *CCR9* C-C chemokine receptor type 9, *HEV* High endothelial venules, *MadCAM-1* Mucosal vascular addressin cell adhesion molecule 1, *VCAM-1* Vascular cell adhesion molecule-1. (Aus Zundler et al. [50] © S. Zundler et al., CC BY 4.0 https://creativecommons.org/licenses/by/4.0/)

Vedolizumab blockiert das Integrin-α4β7 und hemmt die Migration von Immunzellen in den Intestinaltrakt (Abb. 3). Interessanterweise hatte sich in klinischen Studien der reine Integrin-β7-Antikörper Etrolizumab als nicht ausreichend wirksam erwiesen. Natalizumab blockiert als Integrin-α4-Antikörper die Migration von Immunzellen sowohl in den Darm (Integrin-α4β7) als auch ins Gehirn (über Integrin-α4β1), was gelegentlich schwere Komplikationen im Sinne einer progressiven multifokalen Leukenzephalopathie (PML) zur Folge hatte. Das Nebenwirkungsrisiko von Vedolizumab ist sehr günstig, und leichte und schwere UAW wurden lediglich auf Placeboniveau beobachtet [19]. Ein Risiko zerebraler Infektionen (einschließlich PML) scheint bei Vedolizumab nicht zu bestehen. Nasopharyngitis und obere Atemwegserkrankungen sind plausible UAW, denn die vedolizumabvermittelte α4β7-Integrin-Blockade verhindert auch die Einwanderung von Immunzellen in die oberen Atemwege. Durch die darmspezifische Immunsuppression ist Vedolizumab bei EIM weniger wirksam und kann sogar Arthralgien demaskieren.

Vedolizumab ist bei CU und MC zugelassen und war bei CU in einer Head-to-Head(H2H)-Studie Adalimumab überlegen (s. Abschn. „Vergleich der Wirksamkeiten“; [20]). Vedolizumab ist außerdem die fortgeschrittene Therapie der Wahl für die chronische therapierefraktäre Pouchitis, denn die Überlegenheit gegenüber Placebo konnte in einer randomisierten kontrollierten Studie nachgewiesen werden [21].

Die Voraussage des Therapieansprechens ist eine der relevantesten klinischen Forschungsfragen bei CED. Für Vedolizumab bei MC wurde ein einfaches Tool entwickelt: niedriger Albuminspiegel, hohe Konzentration des C‑reaktiven Proteins (CRP) sowie vorausgegangene Anti-TNF-Therapie, gastrointestinale Operationen und fistulierende Erkrankung hatten ein Therapieversagen von Vedolizumab vorausgesagt [22].

#### Merke

- Vedolilzumab hat einen darmspezifischen Wirkungsmechanismus und ein günstiges Nebenwirkungsprofil.
- Vedolizumab ist bei Colitis ulcerosa in einer Head-to-Head Studie einem TNF-Inhibitor hinsichtlich verschiedener Endpunkte überlegen gewesen.

### Interleukin-12/-23- und Interleukin-23-Hemmer

#### Interleukin-12/-23-Hemmer (Ustekinumab)

**Abb. 4:**
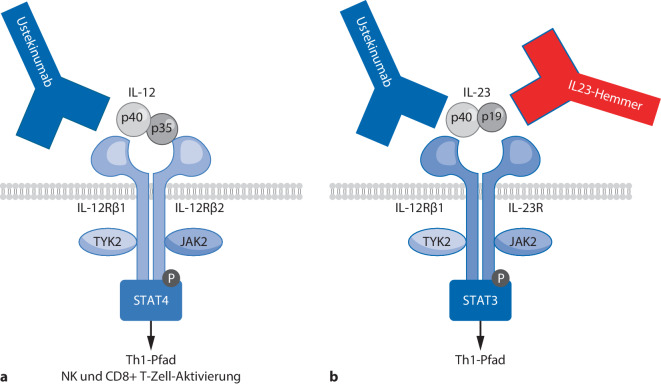
**a, b** Wirkmechanismen von Interleukin(IL)-12/-23 sowie IL-23-Hemmern. Ustekinumab bindet an die gemeinsame Untereinheit p40 von IL-12 und IL-23 und blockiert beide Zytokine. Selektive IL-23 Hemmer binden an p19 und bewirken eine selektive Inhibition von IL-23. *CD* cluster of differentiation, *IL* Interleukin. *JAK2* Januskinase 2, *NK* natürliche Killerzellen, *STAT* signal transducer and activator of transcription, *Th1* T-Helferzellen 1, *TYK2* Tyrosinkinase 2. (Mod. nach [51])

Ustekinumab blockiert die gemeinsame p40-Untereinheit von Interleukin(IL)-12 und IL-23 (Abb. 4) und kann beide Zytokine inaktivieren, mit guten therapeutischen Effekten bei CU und MC. Ustekinumab ist wirksam bei Psoriasis und Psoriasisarthritis, bei einigen EIM (z. B. axiale Arthritis) ist jedoch eine geringere Wirksamkeit als durch TNF-Inhibitoren zu erwarten. Vorteil ist die hohe Sicherheit des Medikaments, denn Nebenwirkungen waren in einer großen Zusammenfassung randomisierter Studien nominal seltener als bei Placebo [23]. Seit 2024 sind für MC Ustekinumab-Biosimilars zugelassen, bei CU behält der Originalhersteller noch das Exklusivrecht durch Patente auf das Dosierungsschema.

##### Merke

- Ustekinumab ist bei MC und CU zugelassen und hat ein günstiges Nebenwirkungsprofil.
- Seit 2024 sind für die MC-Therapie Ustekinumab-Biosimilars zugelassen.

#### Selektive Interleukin-23-Hemmer (Mirikizumab, Risankizumab, Guselkumab)

Die IL-23-Hemmer blockieren die p19-Untereinheit von IL-23 und entfalten eine selektive Wirkung gegen IL-23 ohne Effekte auf IL-12 (Abb. 4b). Risankizumab (MC und CU) und Mirikizumab (CU) wurden von der EMA bereits zugelassen; die Zulassungen von Mirikizumab für MC sowie Guselkumab für MC und CU werden für 2025 erwartet.

Möglicherweise ist die selektive IL-23-Hemmung der IL-12/-23-Hemmung überlegen. Alle IL-23-Hemmer wurden gegenüber dem IL-12/-23-Hemmer Ustekinumab in H2H-Studie verglichen. In der SEQUENCE-Studie mit TNF-erfahrenen MC-Patienten hatte Risankizumab höhere Raten klinischer Remission („non-inferiority endpoint“) und endoskopischer Remission („superiority endpoint“) als Ustekinumab erzielt [24]. Die SEQUENCE-Studie wurde unverblindet durchgeführt (bedingt durch nichtüberlappende Zeitpunkte der Gabe beider Medikamente und den Aufwand, den eine Double-Dummy-Medikation benötigt hätte) und zeigte unterschiedliche Drop-out-Raten in beiden Studiengruppen. Die gehäuften Studienbeendigungen unter Ustekinumabgabe (die in verblindeten Studien nicht beobachtet wurden) könnten durch „non-responder imputation“ zum Unterschied der beiden Substanzen beitragen. Mirikizumab hatte bei MC im verblindeten direkten Vergleich Gleichwertigkeit („non-inferiority“) in den primären und sekundären Endpunkten gezeigt [25], während Guselkumab bei MC bei vielen sekundären Endpunkten Ustekinumab überlegen zu sein scheint [54]. Für Guselkumab wird neben selektiver IL-23-Inhibition auch eine CD64-Bindung diskutiert, mit aktuell unklarer Relevanz und unklarem therapeutischen Nutzen.

Die Medikamentenentwickler haben aus der Erfahrung mit Biologika bei CED gelernt, und Risankizumab und Mirikizumab werden in sehr hohen Dosierungen eingesetzt (Tab. 1). Dies macht die Verwendung eines „On-Body-Injektors“ (Risankizumab) bzw. die Verabreichung von 2 s.c.-Spritzen alle 4 Wochen (Mirikizumab) notwendig. Hohe Dosierungen sind bei CED meist sinnvoll (s. Abschnitt Biologikaspiegel und Anti-Drug-Antikörper); ob das Ergebnis der SEQUENCE-Studie durch die hohe Risankizumabdosis mitbedingt ist, ist offen. Das Nebenwirkungsprofil der IL-23-Hemmer ist gemäß aller verfügbaren Daten sehr günstig und auf Placeboniveau.

##### Merke

- Die IL-23-Hemmer entfalten eine selektive Wirkung gegen IL-23 ohne Effekte auf IL-12.
- Risankizumab (MC und CU) und Mirikizumab (CU) wurden von der EMA bereits zugelassen; die Zulassungen von Mirikizumab für MC sowie Guselkumab für MC und CU werden für 2025 erwartet.
- Das Nebenwirkungsprofil der IL-23-Hemmer ist gemäß aller verfügbaren Daten sehr günstig und auf Placeboniveau.

### Januskinasehemmer (Tofacitinib, Filgotinib, Upadacitinib)

**Abb. 5:**
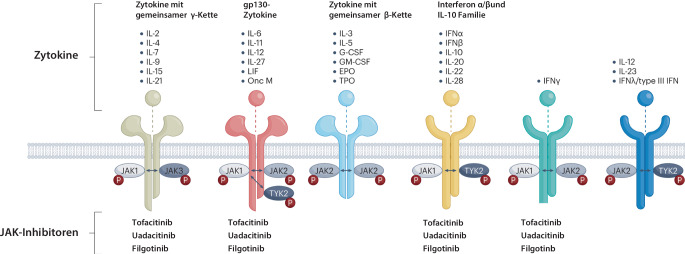
Molekularer Mechanismus der Januskinase(*JAK)*-Inhibitoren. Die JAK bewirken eine Signaltransduktion, die Übertragung des Signals der Zytokine ins Zellinnere. Tofacitinib inhibiert JAK 1 und JAK 3, während Upadacitinib und Filgotinib JAK-1-selektiver sind. Die Abbildung veranschaulicht den breiten Wirkungsmechanismus der JAK mit den Potenzialen von breiter Wirkung und unerwünschten Arzneimittelwirkungen. *EPO* Erythropoetin, *G‑CSF* granulozytenkoloniestimulierender Faktor, *GM-CSF* Granulozyten-Makrophagen-koloniestimulierender Faktor, *IFN* interferon, *IL* Interleukin, *LIF* Leukemia inhibitory factor, *TPO* Thrombopoetin, *TYK2* Tyrosinkinase 2. (Aus Szekanecz et al. [52])

Januskinasen (JAK) sind an der Übertragung der Signale der Zytokine in die Zelle beteiligt (Abb. 5). Der römische Gott Janus hat zwei Gesichter, verkörpert Anfang und Ende und ist der Namensgeber des Monats Januar. Ähnlich wie Janus sind JAK nach zwei Richtungen orientiert, dem Außenraum der Zelle mit vielen Zytokinen und dem Zellinneren, in dem diese Signale umgesetzt werden.

Vier JAK-Inhibitoren (JAK 1–3 und Tyrosinkinase 2 [TYK2]) vermitteln die Signale von vielen Zytokinen, einschließlich IL‑6, IL-12, IL-23, und Interferon‑γ (IFN). Die große Zahl der blockierten Zytokine macht die breite Wirksamkeit der JAK-Hemmer plausibel, mit dem Risiko möglicher Nebenwirkungen. Die JAK-Inhibierung ist dosisabhängig, bei niedriger Dosis blockiert Tofacitinib bevorzugt JAK 1 und JAK 3, während Upadacitinib und Filgotinib eine gewisse Spezifität für JAK 1 zeigen. Die JAK-Inhibitoren zeichnen sich durch eine hohe Wirksamkeit und ein schnelles Therapieansprechen aus. Upadacitinib wird in relativ hohen Dosen eingesetzt, und die Wirksamkeit für CU konnte eindrucksvoll belegt werden [26].

In den Zulassungsstudien bei CED hatte es bei keinem JAK-Inhibitor ernsthafte Sicherheitssignale gegeben [26–29]. Nach Zulassung von Tofacitinib für die rheumatoide Arthritis (RA) wurde gemäß Auflagen der Food and Drug Administration (FDA) für Tofacitinib eine große randomisierte Sicherheitsstudie bei Patienten mit RA durchgeführt (ORAL-Surveillance-Studie). Durch den Einschluss von Hochrisikopatienten (medianes Alter 61 Jahre mit ≥ 1 kardiovaskulärem Risikofaktor) ist die Studie zum Einschätzen der Medikamentensicherheit adäquat gepowert [30]. In der Tofacitinibgruppe (5 mg oder 10 mg) fanden sich signifikant häufiger Malignome (Hazard Ratio [HR] 1,5, Konfidenzintervall [KI]: 1,04–2,09) und ein Trend für eine erhöhte kardiovaskuläre Morbidität (HR 1,33, KI: 0,91–1,94) im Vergleich zu den TNF-Inhibitoren. Weitere Sicherheitssignale in der 10 mg Tofacitinibgruppe betreffen schwere Infektionen, Thrombosen und Mortalität [30]. Aus diesem Grund bestehen aktuell Warnungen von EMA und FDA für alle JAK-Inhibitoren. Insbesondere bei Patienten ≥ 65 Jahre, mit arteriosklerotischen kardiovaskulären Erkrankungen und Risikofaktoren für maligne Erkrankungen bzw. manifesten Malignomen sollten JAK-Inhibitoren nur verwendet werden, wenn keine Therapiealternativen bestehen. Die aktuell vorliegenden Sicherheitsanalysen für Tofacitinib, Filgotinib und Upadacitinib bei CED-Patienten haben ein erwartbares Signal gezeigt für Infektionen, jedoch nicht für die anderen UAW, die in der ORAL-Surveillance-Studie identifiziert wurden [31]. Diese Untersuchungen haben jedoch nicht die Hochrisikopopulation mit der Qualität und der Power der ORAL Surveillance-Studie getestet, daher sind weitere Daten wünschenswert.

Opportunistische Infektionen sind die relevanteste unmittelbare Nebenwirkung der JAK-Inhibitoren, und diesbezügliche Patienteninformation und Impfungen, z. B. gegen Infektionen mit dem Varicella-zoster-Virus sind entscheidend.

Auch bei JAK-Inhibitoren sind die möglichen UAW in Relation zu der relativ hohen Wirksamkeit, dem schnellen Therapieansprechen und der breiten antientzündlichen Wirkung (relevant bei bestehenden EIM) zu sehen, und bei klarer Indikation sollten JAK-Inhibitoren geeigneten Patienten in keiner Weise vorenthalten werden.

#### Merke

- Die JAK-Inhibitoren zeichnen sich durch schnelles Therapieansprechen, relativ hohe Wirksamkeit und breite antientzündliche Wirkung aus. Ihre möglichen UAW sind in Relation zu diesen Effekten zu sehen.
- Bei klarer Indikation sollten JAK-Inhibitoren geeigneten Patienten in keiner Weise vorenthalten werden.

### Sphingosin-1-Phosphat-Rezeptormodulatoren (Ozanimod, Etrasimod)

**Abb. 6:**
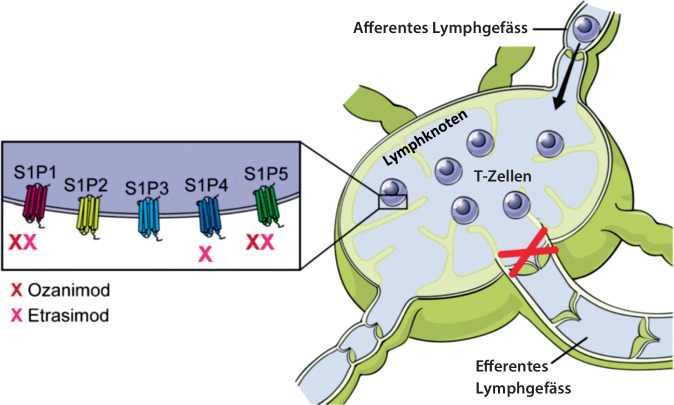
Wirkungsmechanismus der Sphingosin-1-Phosphat-Rezeptormodulatoren. Ozanimod und Etrasimod binden an die Sphingosin-1-Phosphat(*S1P*)-Rezeptoren 1 und 4 bzw. 1, 4 und 5 und bewirken eine Herunterregulierung. Dies hat eine Retention der Lymphozyten im Lymphknoten zur Folge. Direkte Effekte der S1P-Rezeptormodulatoren auf Immunzellen und Darmbarriere sind jedoch ebenfalls relevant. (Aus Shukla et al. [53])

Sphingosin-1-Phosphat(S1P)-Rezeptoren sind breit exprimiert und S1P-Rezeptormodulatoren (S1P-RM) bewirken die verminderte Expression des Rezeptors auf der Zelloberfläche. Ozanimod weist eine Spezifität für die S1P-Rezeptoren 1 und 5 auf, während Etrasimod die S1P-Rezeptoren 1, 4 und 5 inhibiert. Die S1P-RM reduzieren die Migration von Lymphozyten aus den Lymphknoten (was zu einer peripheren Lymphopenie führt), zusätzliche direkte Effekte auf Immunzellen und die intestinale Barriere spielen jedoch ebenfalls eine Rolle (Abb. 6).

Vorteile der S1P-RM sind die einfache Anwendung und die gute Medikamentensicherheit (trotz bzw. aufgrund der unten erwähnten Vorsichtsmaßnahmen). Bei allen Patienten tritt eine (reversible) Lymphopenie auf; diese hängt mit der Medikamentenhauptwirkung zusammen, korreliert aber nicht mit Wirkung und Infektionen (Therapiepause bei absoluter Lymphozytenzahl < 0,2 • 10^9^/l empfohlen). Bei kardial vorbelasteten Patienten (insbesondere bei bradykarden Rhythmusstörungen) sind weitere Vorsichtsmaßnahmen geboten. Zudem sollte bei allen Patienten (Etrasimod immer, Ozanimod bei Risikofaktoren wie einer Vorgeschichte von Diabetes mellitus, Makulaödem und Uveitis) eine ophthalmologische Untersuchung vor bzw. während der Therapie erfolgen.

#### Merke

Vorteile der S1P-RM sind die einfache Anwendung und die gute Medikamentensicherheit (trotz bzw. aufgrund der notwendigen Vorsichtsmaßnahmen).

## Vergleich der Wirksamkeiten

Um die Wirksamkeiten der CED-Therapien im Vergleich zu beurteilen, ist eine größere Zahl von direkten verblindeten Vergleichsstudien mit unterschiedlichen Therapieklassen notwendig, von denen jedoch nur wenige verfügbar sind: Bei TNF-naiven Patienten mit CU war Vedolizumab Adalimumab in verschiedenen Endpunkten überlegen und hatte nach 52 Wochen höhere Raten klinischer Remission und endoskopischer Response erzielt. Unter Adalimumabanwendung war dagegen die steroidfreie Remission häufiger zu verzeichnen [32]. In einer anderen H2H-Studie waren bei Biologika-naiven MC-Patienten die Raten von klinischer Remission bei Ustekinumab und Adalimumab gleich [20].

Da absolute Effekte zwischen randomisierten Studien nicht direkt miteinander verglichen werden können, wurden Netzwerkmetaanalysen, die Unterschiede in Studiendesign und -population korrigieren, verwendet. Diese Technik wurde für den Vergleich von Nebenwirkungen zwischen Tumortherapien entwickelt und dann für Effizienzvergleiche adaptiert. Bei CED werden die Ergebnisse durch die in vielen Studien erfolgende Rerandomisierung erheblich verzerrt und sind mit großer Vorsicht zu interpretieren. In solchen (indirekten) Vergleichen waren für die Remissionsinduktion bei CU Upadacitinib 45 mg, gefolgt von Infliximab, anderen Medikamenten überlegen [33], während bei MC Infliximab, Risankizumab und Upadacitinib höhere Therapieerfolge als andere Substanzen zeigten [34].

Therapiepersistenz ist ein indirektes Maß für die Qualität eines Medikaments, denn (trotz Confounding) ist ein Zusammenhang mit Therapieeffizienz und UAW anzunehmen. In einer Metaanalyse war die Persistenz bei Ustekinumab und Vedolizumab höher als die der TNF-Inhibitoren [35]. Für andere Medikamentenklassen sind die Daten noch abzuwarten.

## Fazit für die Praxis

- Für die Behandlung der chronisch-entzündlichen Darmerkrankungen (CED) stehen 5 Klassen fortgeschrittener Therapien zur Verfügung.
- Es bestehen keine strikten Vorgaben für eine Therapiewahl oder -sequenz. Therapieentscheidungen sollten gemeinsam mit dem Patienten unter Berücksichtigung von Effizienz, Sicherheit, Erkrankungsaktivität, Erfahrungsgrad, klinischer Präsentation (extraintestinale Manifestationen, perianale Erkrankung, individuelle Risiken), Alter, Schwangerschaftswunsch und Applikationsform getroffen werden.
- Fortgeschrittene Therapien sollte früh im Krankheitsverlauf begonnen werden, um bessere Ansprechraten sicherzustellen und Schäden des Magen-Darm-Trakts durch Stenosierungen, Fisteln, Dysplasien und Operationen zu vermeiden.
- Biosimilarität mit dem dadurch bedingten Preisverfall erleichtert den frühen Einsatz von Biologika.
- Unverändert spricht eine relevante Minderheit der Patienten auf jedes individuelle Medikament nicht an, was wahrscheinlich die Heterogenität der Patienten reflektiert und ein sequenzielles Probieren von Therapiealternativen notwendig macht.
- Wir erwarten, dass gezielte Medikamentenkombinationen, neue Medikamentenklassen und/oder Biomarker für das Therapieansprechen die CED-Therapie weiterverbessern werden.
